# Walking sharks cannot beat the heat

**DOI:** 10.1093/conphys/coab035

**Published:** 2021-05-31

**Authors:** Ian A Bouyoucos

**Affiliations:** Department of Biological Sciences, University of Manitoba, 50 Sifton Rd., Winnipeg, Manitoba R3T 2N2, Canada

Ocean warming is a driving force in marine conservation physiology research. Yet, scientists only began to investigate this threat in sharks in the second decade of the 21st century. Since then, researchers around the world have considered the nearly 1200 species of sharks and their kin and have been working to describe biological traits that can be used to predict ‘winners and losers’ of climate change. The epaulette shark (*Hemiscyllium ocellatum*), for example, is renowned for its ability to live in harsh environments and thought to be a clear winner; however, a recent study has singled out ocean warming as this species’—and possibly many others’—kryptonite.

Carolyn Wheeler and her colleagues from the Anderson Cabott Centre for Ocean Life and the Australian Research Council Centre of Excellence for Coral Reef Studies targeted the epaulette shark as a bioindicator—a so-called canary in the coal mine—of ocean warming for sharks. Wheeler and colleagues wondered, if epaulette sharks cannot beat the heat, how will less-tolerant species fare?

The epaulette shark is an egg-laying species (along with ~40% of all sharks and their kin) only found in Australia’s Great Barrier Reef and is thought to be highly resilient to environmental stress. This species is known for its ability to walk out of water between tide pools, its resilience to ocean acidification conditions and its ability to survive for hours without oxygen! However, as an egg-laying species, epaulette shark embryos develop in eggs deposited on the ocean floor that cannot move if water temperatures prove unfavourable. This makes embryos of epaulette sharks—and other egg-laying species—a potential bottleneck in the tolerance of shark populations to ocean warming.

Wheeler and colleagues reared 27 epaulette shark embryos until hatching. Previous research singled out 32°C as `too hot’, where embryos fail to hatch. Knowing this information, the team cleverly tested epaulette sharks at their average summer water temperature (27°C) as well as mid-of-century (29°C) and end-of-century (31°C) predictions with ocean warming to find their pejus (Latin for ‘getting worse’) temperature. The team tracked the embryos’ growth, development and metabolic costs until hatching, and they continued monitoring newly hatched sharks.

Almost every trait that the team measured decreased under end-of-century (31°C) relative to present-day (27°C) temperatures. Notably, embryos reared at 31°C hatched faster, but were smaller and needed to eat sooner than embryos reared at 27°C. Newly hatched epaulette sharks also took twice as long to recover from exercise challenges at 31°C when compared to 27°C. These data led Wheeler and colleagues to conclude the pejus temperature for epaulette shark development and metabolic performance is somewhere between 29°C and 31°C.

Wheeler and colleagues’ findings suggest that epaulette sharks may be living at the tipping point of their temperature tolerance. As a bioindicator species, it may be that ecologically similar species—particularly other tropical, egg-laying shark species—may also be similarly vulnerable to ocean warming. If such species are unable to find cooler habitats or adapt to warmer water, ocean warming could have detrimental impacts on the growth and development of their embryos, which may hold negative consequences for the health of the ecosystems that those species support.

**Illustrations**: Erin Walsh, ewalsh.sci@gmail.com

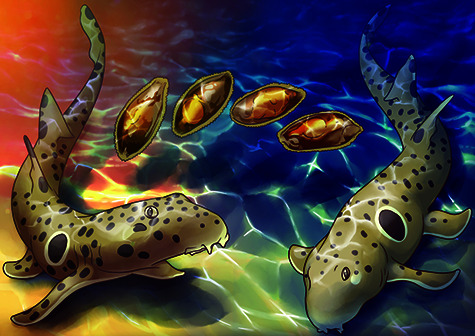



**Editor:** Jodie L. Rummer

## References

[ref1] Wheeler CR, Rummer JL, Bailey B, Lockwood J, Vance S, Mandelman JW (2021) Future thermal regimes for epaulette sharks (*Hemiscyllium ocellatum*): growth and metabolic performance cease to be optimal. Sci Rep 11 (1): 454. doi:10.1038/s41598-020-79 953-0. 10.1038/s41598-020-79953-0 PMC7804200 33436769

